# The Use of Web-Based Patient Reviews to Assess Medical Oncologists’ Competency: Mixed Methods Sequential Explanatory Study

**DOI:** 10.2196/39857

**Published:** 2023-05-04

**Authors:** Nina Morena, Nicholas Zelt, Diana Nguyen, Emilie Dionne, Carrie A Rentschler, Devon Greyson, Ari N Meguerditchian

**Affiliations:** 1 Art History and Communication Studies McGill University Montreal, QC Canada; 2 Faculty of Medicine McGill University Montreal, QC Canada; 3 McGill University Health Centre Research Institute Montreal, QC Canada; 4 St Mary's Research Centre Montreal, QC Canada; 5 School of Population and Public Health University of British Columbia Vancouver, BC Canada; 6 Department of Surgery McGill University Montreal, QC Canada

**Keywords:** web-based patient reviews, medical oncology, cancer care, CanMEDS, RateMDs, web-based physician rating, physician rating websites

## Abstract

**Background:**

Patients increasingly use web-based evaluation tools to assess their physicians, health care teams, and overall medical experience.

**Objective:**

This study aimed to evaluate the extent to which the standardized physician competencies of the CanMEDS Framework are present in web-based patient reviews (WPRs) and to identify patients’ perception of important physician qualities in the context of quality cancer care.

**Methods:**

The WPRs of all university-affiliated medical oncologists in midsized cities with medical schools in the province of Ontario (Canada) were collected. Two reviewers (1 communication studies researcher and 1 health care professional) independently assessed the WPRs according to the CanMEDS Framework and identified common themes. Comment scores were then evaluated to identify κ agreement rates between the reviewers, and a descriptive quantitative analysis of the cohort was completed. Following the quantitative analysis, an inductive thematic analysis was performed.

**Results:**

This study identified 49 actively practicing university-affiliated medical oncologists in midsized urban areas in Ontario. A total of 473 WPRs reviewing these 49 physicians were identified. Among the CanMEDS competencies, those defining the roles of medical experts, communicators, and professionals were the most prevalent (303/473, 64%; 182/473, 38%; and 129/473, 27%, respectively). Common themes in WPRs include medical skill and knowledge, interpersonal skills, and answering questions (from the patient to the physician). Detailed WPRs tend to include the following elements: experience and connection; discussion and evaluation of the physician’s knowledge, professionalism, interpersonal skills, and punctuality; in positive reviews, the expression of feelings of gratitude and a recommendation; and in negative reviews, discouragement from seeking the physician’s care. Patients’ perception of medical skills is less specific than their perception of interpersonal qualities, although medical skills are the most commented-on element of care in WPRs. Patients’ perception of interpersonal skills (listening, compassion, and overall caring demeanor) and other experiential phenomena, such as feeling rushed during appointments, is often specific and detailed. Details about a physician’s interpersonal skills or “bedside manner” are highly perceived, valued, and shareable in an WPR context. A small number of WPRs reflected a distinction between the value of medical skills and that of interpersonal skills. The authors of these WPRs claimed that for them, a physician’s medical skills and competence are more important than their interpersonal skills.

**Conclusions:**

CanMEDS roles and competencies that are explicitly patient facing (ie, those directly experienced by patients in their interactions with physicians and through the care that physicians provide) are the most likely to be present and reported on in WPRs. The findings demonstrate the opportunity to learn from WPRs, not simply to discern physicians’ popularity but to grasp what patients may expect from their physicians. In this context, WPRs can represent a method for the measurement and assessment of patient-facing physician competency.

## Introduction

### Background

Web-based rating tools are prominent across industries, ranging from travel and hospitality to education and health. Their prevalence indicates the importance of reputation management systems in consumer decision-making. In health care, patients use web-based evaluation tools to assess their physicians, health care teams, and overall medical experience. Web-based patient reviews (WPRs) on websites such as RateMDs [1] continue to increase in popularity [2-4] and consist of a rich source of information on patient experience [4,5]. The terms “web-based physician ratings” and “physician rating websites” are also used in the literature to refer to patient-provided ratings and reviews of physicians. Although WPRs may offer information and perspectives valued by other patients, concern has been expressed regarding the representativeness and bias of these perspectives [6,7], and little is known about their potential utility as a source of feedback that might be used for improving care quality.

Web-based health information seeking has a substantial effect on reducing patient uncertainties [8]. WPRs can be considered a valid measure of patient experience, although not necessarily of care quality [9]. Although WPRs are growing in popularity [2], research demonstrates that web-based patient ratings may not be the best indicator of quality health care because of the low correlations between WPR scores and validated survey instruments [10]. Physician demeanor is a dominant factor in both positive and negative reviews [11]. It is challenging to use WPRs to observe changes in physician behavior or performance over time [12]. Patients respond most to certain quality signals, primarily physicians’ interpersonal and clinical skills [13]. Expressions of emotion and disease severity moderate WPR helpfulness levels, and expressions of anger in particular have a negative impact on the perceived helpfulness of physicians, specifically in terms of severe diseases [14]. The average number of physician ratings has almost doubled between 2010 and 2019 [15]. WPRs have a meaningful impact on the selection of one’s physician [16]. Overall, web-based health information seeking has an impact on patients’ evaluation of physicians, as well as on patients’ psychology and behavior [17].

This study draws on the CanMEDS Framework to assess the presence of standardized physician competency in WPRs. The CanMEDS Framework “identifies and describes the abilities physicians require to effectively meet the health care needs of the people they serve” [18]. The main purpose of the Framework is “to define the necessary competencies for all areas of medical practice and provide a comprehensive foundation for medical education and practice in Canada” [19]. The physician abilities described in the CanMEDS Framework are split into 7 roles: medical experts, communicators, collaborators, leaders, health advocates, scholars, and professionals. According to the Royal College, “a competent physician seamlessly integrates the competencies of all seven CanMEDS Roles” [18]. Each role is defined by key concepts, key competencies, and enabling competencies.

The specialty of medical oncology is characterized by rapid and consequential decision-making regarding prognosis and the prescription of highly toxic medications, both of which occur during a period of heightened stress for the patient. Medical oncology requires a combination of skills related to collaboration, communication, and professionalism, ultimately delivering technical and clinical knowledge in practice. A patient’s experience with their medical oncologist is a crucial element of care that often marks the beginning of their cancer journey, as the physician’s knowledge and interpersonal skills have a substantial impact on the patient’s experience [20,21]. Standard assessment tools (eg, written examinations and objective structured clinical examinations) may not be the most effective in evaluating competencies beyond technical skills and knowledge base. Moreover, these standardized assessments typically take place only at the start of the physician’s career, rather than providing periodic feedback as clinicians grow and adapt and as standards and patient expectations change over time. As unstructured and unsolicited assessments, WPRs provided at any time throughout the physician’s career may potentially represent a more meaningful source of ongoing physician evaluation at the experiential level. Altogether, the prevalence of WPRs, the importance of physician demeanor in patients’ perception of care, and the challenges of cancer treatment lead to the following 2 research questions: To what extent do the standardized competencies set out by the CanMEDS Framework appear in the WPRs of medical oncologists? and How do patients, when producing WPRs, perceive and assess the quality of their experience with their medical oncologist?

### Goal of This Study

The purpose of this study was to evaluate the contributions and potential utility of WPRs in the context of medical oncology patient-provider relationships. Given the difficulty of interpreting the extent to which WPRs are valid or reliable measures of physician performance [6], scoring comments according to a valid and established framework provides an opportunity to measure their potential value. The CanMEDS Framework identifies and describes the physician competencies required for the effective delivery of health care. We assessed the presence of CanMEDS competencies in WPRs using a reproducible structure for ranking web-based comments and matching them with specific CanMEDS competencies.

This 2-stage mixed methods analysis of the WPRs of medical oncologists aimed to quantify the focus on specific CanMEDS competencies and to qualitatively describe the contents of such patient-provided assessments of medical competencies. The goal was to understand which CanMEDS competencies are the most frequently commented on in the WPRs of medical oncologists. The outcomes of this study include knowledge of the extent to which CanMEDS competencies are present in WPRs as well as an understanding of patients’ perception of important physician qualities in the context of quality cancer care.

## Methods

This mixed methods study used a sequential explanatory design to identify (1) the extent to which CanMEDS competencies are reflected in WPRs, (2) the most prevalent CanMEDS competencies described in WPRs, and (3) patients’ perceptions of important and reviewable physician qualities in relation to quality cancer care.

### Data Collection

The WPRs of university-affiliated medical oncologists in 4 midsized urban areas with medical schools in the province of Ontario (Canada) were collected for the purpose of this study. Physicians were identified by searching the College of Physicians and Surgeons of Ontario (CPSO) by city, place of practice, and specialty (medical oncology). The CPSO is an organization that “regulates the practice of medicine in Ontario” [22]. As such, its website provides a searchable directory of every physician who is licensed to practice medicine in the province of Ontario, with the goal of helping the population make decisions when seeking a health care provider. The CPSO does not provide information on physicians’ birth dates. The CPSO provides the following information for each physician: name, gender, date of registration and class, languages spoken, education, location of practice, hospital privileges, specialties, terms and conditions, postgraduate training, registration history, and disciplinary details. Toronto (University of Toronto) and Thunder Bay (Northern Ontario School of Medicine) were excluded from this study to ensure even distribution among the cohort. Relative to Toronto, which has a larger population, and Thunder Bay, which has a smaller population, the 4 cities chosen for the study are of a similar size and socioeconomic profile. Ottawa (University of Ottawa), Hamilton (McMaster University), Kingston (Queen’s University), and London (Western University) were the 4 sites that comprised the basis of data collection (Table 1).

**Table 1:** Physicians’ city and institution of primary practice.

City (population density^a^ [km^2^]) and POP^b^			Physicians (N=49), n (%)^c^
**Belleville^d^ (818.8)**			
	Belleville General Hospital	1 (2)	
**Brantford^d^ (1609.0)**			
	Brantford General Hospital	2 (4)	
**Burlington^d^ (987.3^e^)**			
	Joseph Brant Hospital Cancer Clinic	1 (2)	
**Hamilton (1972.4)**			
	Juravinski Hospital	14 (29)	
**Kingston (1573.7)**			
	Cancer Centre of Southeastern Ontario	6 (12)	
**London (1649.3)**			
	London Health Sciences Centre	9 (18)	
**Ottawa (1900.0^f^)**			
	Children’s Hospital of Eastern Ontario	1 (2)	
	Ottawa Hospital Cancer Centre	12 (24)	
	Private practice	2 (4)	
	N/A^g^	1 (2)	

^a^Population density was sourced from Statistics Canada from the 2016 census results, under “population centre” [23].

^b^POP: place of practice.

^c^Number of physicians with that location as their location of primary practice. Percentage represents the percentage of that location’s frequency in our study.

^d^Belleville, Brantford, and Burlington appear on this table because several physicians had more than one location listed as a place of practice.

^e^Population density for Burlington is for the census subdivision; there were no reports for a population center that includes Burlington.

^f^Population density for the Ottawa population center includes the residents of Gatineau (Quebec).

^g^N/A: not applicable.

RateMDs profiles of the cohort were identified based on the CPSO search results. To capture all RateMDs profiles, all possible name permutations were searched, such as the physician’s first name and surname, middle name and surname (if applicable), or only the surname. Launched in 2004, RateMDs is a common platform for patients to post reviews of physicians. WPRs on RateMDs [1] include an average number of stars ranging from 1 to 5, which accounts for the patient’s rating of the staff and their punctuality, helpfulness, and knowledge, as well as written comments. WPRs are posted anonymously and voluntarily (as such, there is no information available on the sociocultural levels of the WPR authors in this study). WPRs consist of a numerical ranking and often include a written comment. WPR authors are asked to select whether the visit they are reviewing occurred in-person, via real-time video, telephone, or other means. They are then prompted to describe their experience through text. The prompt reads, “Please leave a comment with more detail about your experience” [24]. RateMDs provides the following additional information: physician specialty (based on an existing list created by RateMDs), overall specialty numerator and denominator based on ratings (eg, 1 of 200), posting date, and the number of comments marked “helpful” by anonymous readers on the web.

All data in this study were preexisting, and there was no collection of personal information beyond that publicly available in the CPSO database on the web. The reviews of physicians identified as eligible through the CPSO search were downloaded to a spreadsheet. Multiple profiles (eg, using name variations) of the same physician were merged. Reviews were anonymized for reviewer and physician information. Data collection occurred in August 2020.

### Data Analysis

The CanMEDS Framework, established by the Royal College of Physicians and Surgeons of Canada, identifies and describes the physician competencies required for the effective delivery of health care. CanMEDS competencies are divided into the roles of medical experts, communicators, collaborators, leaders, health advocates, scholars, and professionals, with key concepts and enabling competencies constituting the definitions of each role.

This study considers validity not in terms of the correlation between physician reputation and clinical outcomes but between patient evaluation and recognized physician competencies. Validity was established through a matching process. If a WPR reflected one or more CanMEDS roles and could be associated with physician competencies, it achieved validity. The WPR validity increased based on how many roles or competencies were present within the comment. However, all WPRs, independent of their level of association with physician competencies, were considered in the subsequent qualitative analysis to identify themes or qualities that may exceed, depart from, or add nuance to the CanMEDS Framework.

### WPR Assessment and Quantitative Analysis

Two reviewers—1 communication studies researcher and 1 health care professional—independently assessed WPRs according to the CanMEDS Framework and identified common themes using pragmatic analysis [25]. WPRs were collected, anonymized, and exported to a spreadsheet by the research assistant. The reviewers familiarized themselves with the CanMEDS Framework and then matched the WPR text with one or more CanMEDS roles. Once the roles were selected, the reviewers selected the key concepts and enabling competencies that best associated with the WPRs.

The reviewers also took note of cases in which the WPRs mentioned phenomena not specific to CanMEDS, such as punctuality or wait time. In addition to matching the WPR text with the CanMEDS Framework, the reviewers recorded whether the review was positive, negative, mostly positive, mostly negative, physician positive and ancillary negative, physician negative and ancillary positive, or ancillary or other. In this context, ancillary care refers to experiences outside the physician’s care but related to the medical experience, such as those associated with nursing or administrative staff, technicians, directions, parking, traffic, or other obstacles on the way to one’s appointment. Comment scores were then evaluated to identify κ agreement rates between the reviewers, and a descriptive quantitative analysis of the cohort was completed. The WPRs were matched with CanMEDS in December 2020.

### Qualitative Thematic Analysis

Following the quantitative analysis, the WPR text was exported to NVivo (QSR International) for qualitative thematic analysis. An inductive thematic analysis was performed [26]. Portions of the WPR text were coded to nodes that corresponded to the themes. Drawing from López et al [27], the WPR text was also coded to a case to classify the WPR as concise or elaborate depending on the level of detail offered by the author of the WPR. Concise WPRs represented written comments that included little detail but still clearly articulated their author’s experience and perception. Elaborate WPRs are WPRs that tended to include several specific details and often described particular anecdotes. The thematic analysis was performed in October 2021.

The subsequent inductive thematic analysis contributed to the initial deductive approach using the CanMEDS Framework. CanMEDS is a standardized set of documentation; by using it as a reference point, we can identify instances when WPRs directly comment on qualities that physicians are meant to adhere to. Therefore, the subsequent thematic analysis provided knowledge on how patient concerns are expressed through WPRs and the specificities with which they are written. Our inductive approach allowed for additional nuances that could not be provided by the initial analysis. The first analysis revealed which of the CanMEDS roles and competencies are discussed in WPRs, whereas the second revealed how CanMEDS manifests in the written text of WPRs. These approaches fit together because they each offer results the other cannot; therefore, each provide complementary answers to the overarching research questions of the extent to which the CanMEDS roles and competencies are present in WPRs and the ways in which patients perceive and assess physician quality through WPRs.

### Ethical Considerations

All data in this study exist in the public domain; therefore, this study was deemed exempt by the research ethics board of McGill University. WPRs are authored anonymously. WPR author identities cannot be known and are not known by any authors of this paper. The identities of the physicians being reviewed were known only by the first author at the time of data collection. When the first and second authors scored the WPRs, they had no access to the identities of the physicians for whom the WPRs were written. Any WPR that mentioned a physician’s name was deidentified. All deidentification processes were completed by a research assistant.

## Results

### Data Collection

This study identified the RateMDs profiles of 49 actively practicing university-affiliated medical oncologists in midsized urban areas in Ontario, Canada (Table 2). Of the included physician profiles, 71% (35/49) were those of men. All the physicians spoke English. French was the most common second language (13/49, 27%). Overall, 73% (36/49) of the physicians studied at a domestic university, whereas 27% (13/49; 6/13, 46% women and 7/13, 54% men) studied at a foreign university. Furthermore, 31% (15/49) of the physicians obtained their undergraduate degree between 1990 and 1999.

We identified a total of 473 WPRs reviewing these 49 physicians. Of the included physician profiles, 37% (18/49) had 5 to 9 WPRs. The mean word count per physician was 52.15 (SD 23.7) words. Agreement levels between the coders (κ scores) were high in all roles (weighted κ=0.71-1.00). In addition to commenting on physician experience, 20.9% (99/473) of the WPRs contained a mention of the support staff, and 23% (109/473) of the WPRs were clearly identifiable as being written by care providers.

**Table 2:** Physician profiles (N=49).

Characteristics				Physicians, n (%)
**Gender**				
	Men			35 (71)
	Women			14 (29)
**Total number of languages spoken**				
	1			27 (55)
	2			20 (41)
	3			1 (2)
	4			1 (2)
**Languages^a^**				
	English			49 (100)
	French			13 (27)
	Punjabi or Panjabi			2 (4)
	Afrikaans			2 (4)
**Location of medical training**				
	**Domestic**			
		Women (n=14)	8 (57)	
		Men (n=35)	28 (80)	
	**Foreign**			
		Women (n=14)	6 (43)	
		Men (n=35)	7 (20)	
**Year of MD^b^ graduation**				
	Before 1980			8 (16)
	1980-1989			14 (29)
	1990-1999			15 (31)
	2000-2009			11 (22)
	2010-2019			1 (2)

^a^All the other languages had only 1 count. Other languages included Bengali, Cantonese, Czech, German, Hindi, Romanian, Spanish, and Urdu.

^b^MD: Doctor of Medicine.

### WPR Assessment and Quantitative Analysis

Among the CanMEDS competencies, those defining the roles of medical experts, communicators, and professionals were the most prevalent (303/473, 64%; 182/473, 38%; and 129/473, 27%, respectively). The identified themes were similar between positive and negative evaluations. The most-discussed positive themes were knowledge and compassion (Table 3). Less prominent positive themes pertained to follow-up quality, referral, family relationship, current knowledge, and research participation. The most common negative theme was poor interpersonal skills. Negative themes also pertained to lateness, punctuality, or overall wait time for appointments. As expected, negative comments tended to represent the opposite of the most common positive themes, such as a lack of compassion or communication skills, a perceived lack of knowledge, and an unprofessional behavior. Overall, 38.1% (180/473) of the WPRs were marked as helpful by anonymous readers, and 27.9% (132/473) of the WPRs provided a high (4-5 stars out of 5) rating.

**Table 3:** Comment themes by the CanMEDS roles.

Themes	CanMEDS role	Key concepts, n	Competencies, n	Key concepts, wκ^a^ (SD)	Competencies, κ^b^ (SD)
Knowledge^c^	Medical expert	20	17	0.71 (0.16)	0.86 (0.10)
Compassion^d^	Communicator	24	17	0.80 (0.12)	0.88 (0.09)
Effective referral	Collaborator	18	7	0.98 (0.08)	0.99 (0.05)
N/A^e,f^	Leader	22	17	0.99 (0.04)	1.00 (0.02)
N/A^e,f^	Health advocate	15	6	1.00 (0.03)	1.00 (0.02)
Personal learning or research	Scholar	44	18	0.98 (0.02)	1.00 (0.02)
Compassion or relationship with family	Professional	29	13	0.93 (0.06)	0.93 (0.06)

^a^wκ: Weighted kappa: weighted by number of competencies.

^b^κ: Unweighted, unstratified kappa.

^c^Includes compassion and follow-up care.

^d^Includes listening, explaining, relationship with family, and feelings of being rushed (or not).

^e^N/A: not applicable.

^f^No consistent themes were identified.

### Qualitative Thematic Analysis

The inductive thematic analysis revealed the themes in Table 4. The percentages listed refer to the amount of coded text in relation to all WPR text. All themes included WPRs that were considered either concise or elaborate. In total, 78% (369/473) of the comments were coded as concise, and 19% (90/473) were coded as elaborate.

This section includes excerpts from the WPRs that correspond with the themes of interpersonal skills, medical skill and knowledge, and medical-personal duality, with general and specific examples of each. The first 2 themes represent the 2 most common themes identified, whereas medical-personal duality, a less common theme that combines the interpersonal and medical skills, provides insight into what patients interpret to be the most important elements of care. Concise WPRs regarding interpersonal skills included comments such as “He is very friendly and approachable” and “She can be negative and non-supportive.” Elaborate and interpersonal-themed WPRs described the physician’s communication skills and personality in greater detail:

What a wonderful doctor! She actually listens intensely; changed my treatment regimen because of what I told her. Compassionate, easy to talk to. Very patient-centered.

We found him arrogant, unwilling to share information, unorganized, unprepared, condisending, self centred, distant, uncaring, more willing to talk about his black belt and vacations. The patient was treated as they were a number/file, not someone with a life threatening disease!!

Regarding medical skill and knowledge, concise WPRs included “extremely knowledgable,” “Knows his stuff,” and “Great oncologist!” Elaborate WPRs that discussed medical skill and knowledge tended to comment on clinical trials, treatment decisions, and prolonged life rather than particular scientific or technical competence:

She kept me alive for 3 years until I became eligible for a clinical trial that is working well for me. She is a very caring compassionate physician, was always looking for new treatment options when my treatments weren’t going well. She even called me at my cottage before she left on her vacation to set up a radiation appt for me.

He has taken all needs into consideration, from physical to emotional to financial. He seems to be very current and informed, and has indicated different studies I could be part of.

Less frequent were the WPRs that considered the balance between medical knowledge and personality. Generally, these WPRs would mention a strength in one and a lack in the other:

Knowledge may be adequate, but overshadowed by significant lack of empathy, leading to lack of confidence in MD’s ability.

Amazing Doctor. We do not need someone to hold our hand, he is brilliant and he is on the cutting edge of cancer treatment.

**Table 4:** Themes identified in the web-based patient reviews (WPRs).

Theme	WPR (N=473), n (%)	Concise	Elaborate
Medical skill and knowledge	386 (27)	“He is knowledgeable, professional, compassionate, positive.”	“I am very glad that I made the effort to put my emotions aside and trust in his knowledge and skill. There is no one better suited to my complex case. We meet on a regular basis, he always gives me the impression he’s just read my file and knows my case in detail, we discuss (dare I say collaborate) my care, and he patiently answers my many questions—even the kooky ‘I just read this on the Internet’ variety.”
Interpersonal skills	372 (24)	“He is very friendly and approachable.”	“Compassionate and patient. My mom always felt at ease during her apps with him-even when news always wasn’t good. He was always positive and would always try his best to find a solution.”
Answering questions	160 (10)	“Very patiently answers every question.”	“If I didn’t understand she would draw pictures to make me see what she was talking about.”
Feeling fortunate or grateful for care	164 (10)	“So lucky to have her work with me.”	“But rest assured, [name], will never give up looking for the best options for his patients. [name] Thanks for being so wonderful for my mom (and dad). She was always thankful you were her doctor. Mom was with us longer and even enjoyed a trip to Cuba -because of your care.”
Medical-personal duality	38 (8)	“I find her to be very very knowledgable and I trust this implicitly. She is a little blunt though and relies and believes statistics too much. She can be negative and non-supportive. I do like though that she understands the disease and is very competent.”	“[Name] is fairly knowledgeable, however his bedside manner can only be described as poor. With the exception of my first appointment, the average length of each of my visits has maybe been 4 minutes. These almost always end with [name] creeping out door while I’m in mid-sentence. He’s impatient, unhelpful and inflexible, especially regarding surveillance, but nonetheless has the potential to become a good practitioner. Ultimately, I referred myself to someone else.”
History	128 (6)	“patient since 2007 when i was diagnosed with breast cancer.”	“I was diagnosed with a brain tumour classified as a diffuse astrocytoma last year. I am still undergoing treatment but so far I have been through brain surgery to debulk most of the tumour, 30 days of targeted radiation to the brain concurrent with daily chemotherapy. Then an additional 6 months of chemo therapy, all of this following a stage 4 protocol.”
Institution	106 (6)	“When you call his office, his staff do a great job in communicating issues.”	“Unfortunately any future questions or requests for an appointment or change to therapy must go through the [City] Hospital Cancer Patient Support Line which is very busy with long wait times. This is why the poor staff rating. Actual staff I spoke with were excellent. When I finally reached the patient support line the nurse indicated she would contact me as to whether [name] could modify the prescription—this did not happen and I never knew it was done (despite a repeat call to the Patient Support Line) until I called the pharmacy several weeks later to check.”
Punctuality	77 (4)	“Always kept waiting quite late for each appointment.”	“As far a punctuality goes, people should learn a little patience. We never know what kind of news a doctor has to give a patient and I know from experience that I would rather have a doctor who spent 5 or 10 minutes longer than the alloted time for an appointment than having a doctor who rushes you off without explaining what is happening to you.”
Recommendation	66 (3)	“If you are in [name]’s hands you are one lucky patient!!”	“If you find you are his patient, or family or friends of his patient, then please understand that you are strong, and do not let his discomfort and anxiety spread to you. Furthermore, you are always permitted, and it is recommended, to get other opinions and change to another oncologist.”
Organization of care	23 (2)	“He even returns phone calls promptly if needed!”	“Refused to let me tape the appointment. What does he have to hide? Examines female patients without a nurse present. Also asks that the person the patient brings with them not be in the room, which is beyond inappropriate. If a patient wants someone present it is not for the doctor to decide. This is against [Hospital] policy.”
Feeling rushed	53 (2)	“I never felt like I was taking too much of their time.”	“[name] is rushed at every visit. It feels like his thoughts are already onto the next patient. He is in a race with himself to see how many words he can speak per minute to hell with what the patient is thinking or feeling as he spu’s the information at you.”
Being busy	16 (1)	“Unfortunately he’s very busy and doesn’t seem to have sufficient time to be able to spend with each patient.”	“He was awful and unkind and made it clear that he was very busy and had more important things to do than speak to me.”

In more specific terms, the following WPR offers a description of both the physician’s interpersonal skills and medical expertise. The author interprets what may be perceived as a lack of compassion as evidence of the physician’s increased attention to detail and competence in cancer care:

My sister ultimately lost her battle with stage 4 colon cancer, but [name] and his work on clinical trials gave her an extra 2 years. While I agree that he may come across as arrogant and non-empathetic (i remember being horrified after the first meeting), after spending 2 years with the man I can tell you that he has a tough job that none of us could ever imagine doing, and his passion in life is helping people and finding a cure. While his bedside manner could maybe use a little help, he does care, is a great doctor, and if you think he's not present at your appointment, it's because he's busy thinking two steps ahead for you. Brilliant oncologist, [city] is lucky to have him.

Although WPRs do evaluate characteristics found in the CanMEDS Framework, they offer detailed experiences that illustrate how the CanMEDS roles function in practice and are perceived by patients, thereby contributing to our understanding of the significance of physician assessment tools. The themes, stories, details, and patterns revealed by attending to the WPRs qualitatively provided the following insights: (1) confirmation that patients evaluate characteristics found in the CanMEDS Framework, (2) descriptions of how the CanMEDS roles manifest in practice as perceived by patients, and (3) patients’ perceptions of important and expected physician qualities in cancer care. These results indicate that the CanMEDS roles and competencies that are explicitly patient facing (ie, those that are directly experienced by patients in their interactions with physicians and through the care physicians provide) are the most likely to be present and reported on in WPRs.

## Discussion

### Principal Findings

This study uses both quantitative and qualitative methods to assess WPRs as a potential source of information on physician competency using a validated framework such as CanMEDS. To date, studies of WPRs were limited by large data sets spanning several specialties and a focus on numerical star ratings. This work differs because of its focus on a defined subset of physicians within a particular specialty. While the CanMEDS Framework was created for physicians practicing in Canada and has been used in this study to assess the WPRs of physicians in a Canadian context, its competencies represent qualities that are standard and commonplace in medicine, making our observations transposable to other clinical practice contexts. As such, similar assessments could be performed in other countries using the CanMEDS Framework or an equivalent set of guidelines as a reference point.

We found that most RateMDs WPRs of medical oncologists address a limited number of themes, several of which speak directly to CanMEDS competency areas and indicate what patients consider to be important, reportable, and shareable qualities within the context of cancer care. Our findings suggest that WPRs may serve as an additional tool for evaluating physician competence, may be relevant to physician training or continuing development, and may be used to flag or prevent lapses in care. In sum, WPRs emphasize experiential competencies related to communication and interpersonal skills and suggest an alternative format for evaluating the care provided by medical oncologists.

### Theoretical Implications

Studies of WPRs tend to be quantitative. Previous studies of web-based evaluations have used methods such as word frequency, surveys, and regression models. These studies were retrospective, observational, and comparative (for instance, comparing data from 2 sources, such as physician peer reviews or institutional reviews with patient-provided web-based reviews). Qualitative studies of WPRs include the use of interviews and content analysis. Many studies of WPRs draw on large cohort sizes, often comprising thousands of web-based reviews. Moreover, research shows that WPRs consider not only doctor-patient encounters but also ancillary forms of care, such as those provided by support staff [27], and the context of care. Urgent care reviews tend to mention operational elements such as staff competence [28]. Moreover, the validity of patient ratings (in terms of their consistency with physician peer reviews) varies by medical specialty [29]. Physician-based and office-based web-based physician reviews do not necessarily correlate highly with each other [30]. For example, Quinones et al [31] also found that discussions of pain management were present in both positive and negative WPRs of neurosurgeons; top-rated WPRs demonstrated the importance of compassion for levels of patient satisfaction. The study by Seltzer et al [32] on patient experiences in web-based reviews of obstetric care revealed that narratives found in WPRs related to obstetrics provide meaningful information toward the improvement of obstetric care experiences.

Our focus on a local or midsized cohort allowed us to manually analyze individual comments and match them with the CanMEDS competencies to evaluate their meaning and importance. By reducing the scale, we have the capacity to test this study’s assessment methods within a relatively homogeneous cohort of physicians, allowing for comparison, which, in future research, can be applied to cohorts of different sizes or characteristics. By matching the WPR text to specific CanMEDS competencies, we were able to identify what competencies could potentially be captured by WPR authors. Most physicians in our cohort were trained, passed certification examinations, and now practice in a Canadian context where the CanMEDS Framework is in effect. Therefore, it is reasonable to expect that they would meet the competencies under which they are expected to practice.

As demonstrated by scholarship on physician ratings, communication and respect between physicians and patients are important elements of patient care [33]. Little is known about the written text that accompanies WPRs [34]. The results of our thematic analysis respond to this gap and suggest that WPRs, when detailed, tend to include the following elements: (1) experience and connection, including information regarding the author of the WPR or their family or friend and the type of cancer and a timeline indicating how long they have been the physician’s patient; (2) discussion and evaluation of the physician’s knowledge, professionalism, interpersonal skills (often described as “bedside manner”), and punctuality; (3) in the case of positive reviews, the expression of feelings of gratitude and thankfulness; and (4) in the case of positive reviews, a recommendation or in the case of negative reviews, discouragement from seeking the physician’s care.

### Practical Implications

The WPRs in this study reflect that patients value time management, access to information, and whole person care or comprehensive care. In this context, time management refers to instances when patients do not feel rushed during their appointments and do not feel as if they are asking too many questions or taking up too much of the physician’s time. Access to information comprises patients being able to receive clear answers to all of their questions or concerns and not being condescended to for raising them. Comprehensive care refers to patients feeling that their physician has taken a genuine interest in them and that they are treated as a whole person and not as a number or disease. Taken together, these are especially valuable and important elements of care in the context of medical oncology, where patients experience pain and side effects as a result of toxic medications and may be overwhelmed with new information.

Although patients’ perception of medical skills is less specific than their perception of interpersonal qualities, medical skills are the most commented-on element of care in the RateMDs WPRs assessed in this study. Patients’ perception of interpersonal skills (listening, compassion, and overall caring demeanor) and other experiential phenomena, such as feeling rushed during appointments, is often specific, detailed, and described with more nuance than what is offered by the CanMEDS Framework. The findings of this study indicate that patients are highly perceptive, particularly of their physician’s interpersonal skills. Patients recognize and describe in WPRs instances when their physician does not remember them, seems unprepared, is not thorough in their examination or questions, and does not seem to care about or value them. This suggests that details about a physician’s interpersonal skills or “bedside manner” are highly perceived, valued, and shareable in a WPR context such as RateMDs.

In addition, we found that only a small number of WPRs reflected a distinction between the value of medical skills and that of interpersonal skills. The authors of these WPRs claimed that, for them, a physician’s medical skills and competence are more important than their interpersonal skills (ie, being a “good doctor” is more important than whether they are polite). Although not representative of most WPRs, this is an important perception and articulation of what qualities matter the most in one’s medical oncologist. However, compassion and politeness remain highly appreciated qualities. Owing to the subjectivity of WPRs, there is an ongoing debate on how the information these evaluations provide should be measured, interpreted, and potentially applied to the improvement of health care. The prevalence of WPRs that evaluate interpersonal communication skills reveals the subjective and experiential nature of what WPRs discuss, part of which may not be testable using standardized assessment tools. Although WPRs provide insight into the patient-doctor relationship, their importance in relation to the quality of health care is understudied [35,36]. Research indicates that the validity of WPRs varies according to medical specialization [29]. For instance, medical practitioners in obstetrics and gynecology face a high risk of patient complaint [37]. It is unclear whether WPRs have enforceable legal value, although they have appeared in lawsuits before, for instance, in cases of libel where a physician considered their WPRs to be defamatory [38,39].

### Interpreting the Significance of WPRs

Patients who leave reviews tend to be women with postsecondary education who have a chronic illness [40]; however, demographic variables and health status do not act solely as predictors for a patient’s use of physician evaluation tools. WPR websites such as RateMDs [1] have a substantial influence on patients who read or write reviews [16,39]. For example, Emmert et al [35] demonstrated that 65% of survey respondents went to a physician based on that physician’s WPRs, whereas 52% did not go to a physician because of their WPRs. Similarly, Li et al [16] demonstrated that patients were considerably more likely to be swayed by web-based ratings than by objective report cards when selecting a cardiac surgeon. Although not completely indicative of health care quality, our findings signify that WPRs are a strong indicator of experiential qualities, which themselves may influence care, particularly in terms of trust and communication between the patient and the physician, and may represent opportunities for feedback and improvement.

Importantly, WPRs exist within a wider environment of sponsorships and reputation management systems. Physicians have the option to pay for additional features on their RateMDs pages, such as banner advertisements for their profiles on other physicians’ profiles [41]. Patients are more likely to share the experiences they had with physicians who have “a higher medical quality and service attitude and who work in hospitals with a higher online reputation” [42]. Social influence, in the form of credibility and performance expectancy, has, at minimum, an indirect impact on the “behavioral intention” to use physician rating websites [43].

Overall, there is consensus that WPRs are important and comprise a valuable source of data, although there is less consensus on how the importance and validity of these ratings can be measured and interpreted to better understand their meaning. Although WPRs continue to grow in popularity, they constitute a minimal proportion of the physician’s actual patients [44]. Moreover, WPRs have been shown to have a poor association with the physician’s clinical performance scores [6]. Qualitative analysis can help validate rating tools [45], as the information provided by WPRs is most valuable when considered in the appropriate context [46]. Therefore, although the available WPRs do consist of valuable data, their small sample size is considered to decrease their overall validity [2,3,47]. It is also recommended that WPRs not be published until a sufficient number of reviews are reached, such as 15 reviews [47]. Outside health care, rating systems have a major impact: if an Uber (Uber Technologies, Inc) driver’s star rating (provided by passengers) drops below the established average for that city, the driver will lose temporary or permanent access to the Uber platform and will not be allowed to drive for Uber [48]. Altogether, the benefits afforded by WPRs, which include a sense of patient empowerment and the perception of informed decision-making, must be considered alongside their flaws, which include a small sample size and poor association with clinical outcomes [6,49]. As shown by our study, WPRs can potentially be used to assess experiential qualities while remaining aware of the possible drawbacks of relying on anonymous web-based content.

### Limitations

The limitations of this study include a cohort of physicians skewed toward men, uneven numbers of WPRs per physician, and the unavailability of the demographic data of the WPR authors. However, the skewed physician gender distribution in this study is representative of the actual gender disparities in medical oncology within the area in which the study cohort was built [50]. The number of WPRs a particular physician has is partially dependent on the number of years the physician has been practicing, which suggests that physicians with more years of experience will have amassed more WPRs than those with fewer years of experience. However, the impact of WPRs on modulating physician behavior remains unknown. Moreover, although this study moves toward an analysis of patient expectations, further analysis using the expectancy-disconfirmation paradigm would be necessary to sufficiently conclude on patient expectations within this study’s results [51].

### Future Research Perspectives

This study gestures toward the potentially valuable content found in WPRs. It demonstrates the opportunity to learn from WPRs, not simply to discern physician popularity but to grasp what patients may expect from their physicians. In this context, WPRs could potentially represent a method for assessing patient-facing physician competencies. The next steps include conducting a survey to determine patients’ own assessment of this study’s results and patient engagement with WPRs in the context of medical oncology, as well as further analysis to identify patient expectations. Future research is required to assess WPRs’ role in and influence on cancer care, medical education, and experiential competency assessment.

## Abbreviations

CPSO: College of Physicians and Surgeons of Ontario
WPR: web-based patient review

## Data Availability

The data sets generated during or analyzed during this study are not publicly available to preserve physician anonymity but are available from the corresponding author upon reasonable request.

## References

[1] RateMDs.

[2] Gao GG, McCullough JS, Agarwal R, Jha AK (2012). A changing landscape of physician quality reporting: analysis of patients' online ratings of their physicians over a 5-year period. J Med Internet Res.

[3] Segal J, Sacopulos M, Sheets V, Thurston I, Brooks K, Puccia R (2012). Online doctor reviews: do they track surgeon volume, a proxy for quality of care?. J Med Internet Res.

[4] Wallace BC, Paul MJ, Sarkar U, Trikalinos TA, Dredze M (2014). A large-scale quantitative analysis of latent factors and sentiment in online doctor reviews. J Am Med Inform Assoc.

[5] Agarwal AK, Pelullo AP, Merchant RM (2019). "Told": the word most correlated to negative online hospital reviews. J Gen Intern Med.

[6] Daskivich TJ, Houman J, Fuller G, Black JT, Kim HL, Spiegel B (2018). Online physician ratings fail to predict actual performance on measures of quality, value, and peer review. J Am Med Inform Assoc.

[7] Huber SA, Priestley J, Kasabwala K, Gadidov B, Culligan P (2019). Understanding your online ratings: a methodological analysis using urogynecologists in the United States. Female Pelvic Med Reconstr Surg.

[8] Dong W, Lei X, Liu Y (2022). The mediating role of patients' trust between web-based health information seeking and patients' uncertainty in China: cross-sectional web-based survey. J Med Internet Res.

[9] Placona AM, Rathert C (2022). Are online patient reviews associated with health care outcomes? A systematic review of the literature. Med Care Res Rev.

[10] Chen J, Presson A, Zhang C, Ray D, Finlayson S, Glasgow R (2018). Online physician review websites poorly correlate to a validated metric of patient satisfaction. J Surg Res.

[11] Pham JT, Kim JK, Hunt SE, Willette DM, Tang CJ (2022). Online patient reviews of breast reconstruction: RealSelf analysis. Plast Reconstr Surg Glob Open.

[12] Wang W, Luo J, Dugas M, Gao GG, Agarwal R, Werner RM (2022). Recency of online physician ratings. JAMA Intern Med.

[13] Chen Y, Lee S (2022). EXPRESS: user-generated physician ratings and their effects on patients’ physician choices: evidence from yelp. J Mark.

[14] Shah AM, Lee KY (2021). The role of emotions intensity in helpfulness of online physician reviews. Intell Autom Soft Comput.

[15] Emmert M, McLennan S (2021). One decade of online patient feedback: longitudinal analysis of data from a German physician rating website. J Med Internet Res.

[16] Li X, Chou SY, Deily ME, Qian M (2021). Comparing the impact of online ratings and report cards on patient choice of cardiac surgeon: large observational study. J Med Internet Res.

[17] Luo A, Qin L, Yuan Y, Yang Z, Liu F, Huang P, Xie W (2022). The effect of online health information seeking on physician-patient relationships: systematic review. J Med Internet Res.

[18] (2023). CanMEDS framework. Royal College of Physicians and Surgeons of Canada.

[19] (2023). About CanMEDS. Royal College of Physicians and Surgeons of Canada.

[20] Mavis B, Vasilenko P, Schnuth R, Marshall J, Jeffs MC (2005). Female patients' preferences related to interpersonal communications, clinical competence, and gender when selecting a physician. Acad Med.

[21] Asanad K, Parameshwar PS, Houman J, Spiegel BM, Daskivich TJ, Anger JT (2018). Online physician reviews in female pelvic medicine and reconstructive surgery: what do patients really want?. Female Pelvic Med Reconstr Surg.

[22] (2023). What we do. College of Physicians and Surgeons of Ontario.

[23] (2017). Census profile, 2016 census. Statistics Canada.

[24] (2023). Doctor reviews and ratings. RateMDs.

[25] Barbour RS, Flick U (2014). Quality of data analysis. The SAGE Handbook of Qualitative Data Analysis.

[26] Braun V, Clarke V, Cooper H, Camic PM, Long DL, Panter AT, Rindskopf D, Sher KJ (2012). Thematic analysis. APA Handbook of Research Methods in Psychology. Research Designs: Quantitative, Qualitative, Neuropsychological, And Biological. Volume 2.

[27] López A, Detz A, Ratanawongsa N, Sarkar U (2012). What patients say about their doctors online: a qualitative content analysis. J Gen Intern Med.

[28] Hu D, Liu CM, Hamdy R, Cziner M, Fung M, Dobbs S, Rogers L, Turner MM, Broniatowski DA (2021). Questioning the Yelp Effect: mixed methods analysis of web-based reviews of urgent cares. J Med Internet Res.

[29] McGrath RJ, Priestley JL, Zhou Y, Culligan PJ (2018). The validity of online patient ratings of physicians: analysis of physician peer reviews and patient ratings. Interact J Med Res.

[30] Zhao HH, Luu M, Spiegel B, Daskivich TJ (2020). Correlation of online physician rating subscores and association with overall satisfaction: observational study of 212,933 providers. J Med Internet Res.

[31] Quinones A, Tang JE, Vasan V, Li T, Schupper AJ, Ali M, White CA, Hannah TC, Asfaw Z, Li AY, Durbin J, Arvind V, KIm JS, Choudhri T (2022). Trends in online patient perspectives of neurosurgeons: a sentiment analysis. Neurosurgery Open.

[32] Seltzer EK, Guntuku SC, Lanza AL, Tufts C, Srinivas SK, Klinger EV, Asch DA, Fausti N, Ungar LH, Merchant RM (2022). Patient experience and satisfaction in online reviews of obstetric care: observational study. JMIR Form Res.

[33] Quigley DD, Elliott MN, Farley DO, Burkhart Q, Skootsky SA, Hays RD (2014). Specialties differ in which aspects of doctor communication predict overall physician ratings. J Gen Intern Med.

[34] Liu C, Uffenheimer M, Nasseri Y, Cohen J, Ellenhorn J (2019). "But His Yelp Reviews Are Awful!": analysis of general surgeons' Yelp reviews. J Med Internet Res.

[35] Emmert M, Meier F, Pisch F, Sander U (2013). Physician choice making and characteristics associated with using physician-rating websites: cross-sectional study. J Med Internet Res.

[36] Hong YA, Liang C, Radcliff TA, Wigfall LT, Street RL (2019). What do patients say about doctors online? A systematic review of studies on patient online reviews. J Med Internet Res.

[37] Spittal MJ, Bismark MM, Studdert DM (2019). Identification of practitioners at high risk of complaints to health profession regulators. BMC Health Serv Res.

[38] Duffy A (2019). Kingston doctor wins cyber libel case against malicious web critic. Ottawa Citizen.

[39] Chiruvella V, Guddati AK (2021). Cyberspace and libel: a dangerous balance for physicians. Interact J Med Res.

[40] Terlutter R, Bidmon S, Röttl J (2014). Who uses physician-rating websites? Differences in sociodemographic variables, psychographic variables, and health status of users and nonusers of physician-rating websites. J Med Internet Res.

[41] Crowe K (2018). Who's rating doctors on RateMDs? The invisible hand of 'reputation management'. Canadian Broadcasting Corporation.

[42] Wang Y, Wu H, Lei X, Shen J, Feng Z (2020). The influence of doctors' online reputation on the sharing of outpatient experiences: empirical study. J Med Internet Res.

[43] Guetz B, Bidmon S (2022). The impact of social influence on the intention to use physician rating websites: moderated mediation analysis using a mixed methods approach. J Med Internet Res.

[44] Syed UA, Acevedo D, Narzikul AC, Coomer W, Beredjiklian PK, Abboud JA (2019). Physician rating websites: an analysis of physician evaluation and physician perception. Arch Bone Jt Surg.

[45] Kadry B, Chu LF, Kadry B, Gammas D, Macario A (2011). Analysis of 4999 online physician ratings indicates that most patients give physicians a favorable rating. J Med Internet Res.

[46] Murphy GP, Radadia KD, Breyer BN (2019). Online physician reviews: is there a place for them?. Risk Manag Healthc Policy.

[47] Okike K, Uhr NR, Shin SY, Xie KC, Kim CY, Funahashi TT, Kanter MH (2019). A comparison of online physician ratings and internal patient-submitted ratings from a large healthcare system. J Gen Intern Med.

[48] (2021). Uber community guidelines: United States and Canada. Uber.

[49] Trehan SK, Daluiski A (2016). Online patient ratings: why they matter and what they mean. J Hand Surg Am.

[50] (2020). Supply, distribution and migration of physicians in Canada, 2020. Canadian Institute for Health Information.

[51] Oliver RL (2018). A cognitive model of the antecedents and consequences of satisfaction decisions. J Mark Res.

